# Dietary factors and polymorphisms in vitamin D metabolism genes: the risk and prognosis of colorectal cancer in northeast China

**DOI:** 10.1038/s41598-017-09356-1

**Published:** 2017-08-18

**Authors:** Chen Gong, Zhiping Long, Yanming Yu, Lin Zhu, Jingshen Tian, Shuo Li, Jing Li, Hongyuan Yu, Qiang Chi, Daxun Piao, Fan Wang, Yashuang Zhao, Binbin Cui

**Affiliations:** 1 Department of Epidemiology, School of Public Health, Harbin Medical University, Harbin, Heilongjiang Province P. R. China; 2 The Third Affiliated Hospital of Harbin Medical University, Harbin, Heilongjiang Province P. R. China; 3 The Second Affiliated Hospital of Harbin Medical University, Harbin, Heilongjiang Province P. R. China; 4 The First Affiliated Hospital of Harbin Medical University, Harbin, Heilongjiang Province P. R. China

## Abstract

*CYP24A1* and *CYP27B1* are critical genes determining 1α,25(OH)_2_D_3_ concentration and impacting on carcinogenesis. A case–control study including 528 colorectal cancer (CRC) patients and 605 cancer-free controls and a follow-up study with 317 cases were conducted in northeast China. Genotypes were tested by TaqMan Genotyping Assays. Individuals carrying the GG genotype of *CYP27B1* G > T (rs10877012) exhibited decreased CRC risk compared with those with the TT genotype (OR_adjusted_ (OR_adj_) = 0.57, 95% Confidence Interval (CI) = 0.38–0.84). Compared with the TT genotype, a significant association between the CC genotype of *CYP27B1* C > T (rs4646536) and a reduced risk of CRC was observed (OR_adj_ = 0.59, 95% CI = 0.40–0.88). We also observed significant combined effects of the two polymorphisms in *CYP27B1* with dietary factors, including the intake of cereals, overnight meal, allium vegetables, pork, canned fruit, and braised fish, on CRC risk. These associations remained significant after Bonferroni correction for multiple comparisons. The Hazard Ration (HR) of patients with the AA genotype (*CYP24A1* A > G, rs4809957) was 2.38 (95% CI = 1.30–4.37) when compared with the GG genotype. Thus, our findings suggested that two polymorphisms in *CYP27B1* are associated with CRC susceptibility. *CYP24A1* A > G (rs4809957) polymorphism may lead to a worse prognosis of CRC.

## Introduction

Colorectal cancer (CRC) is a major public health issue, being the third most common cancer and the fourth most prominent cause of cancer death worldwide. The World Health Organization reported that 253,427 new cases of CRC and 139,416 deaths occurred in China in 2012^1–3^. Suspected or established risk factors of CRC include red meat, alcohol drinking, obesity, physical inactivity, and smoking^1, 4^. In addition to the traditional dietary factors (fibre, fresh fruit and vegetables) that are protective against CRC^5, 6^, a number of studies have suggested that vitamin D contributes to a reduced risk of this disease^7–9^.

More than 90% of vitamin D that the human body needs is obtained from the conversion of 7-dehydrocholesterol to vitamin D_3_, which occurs in the skin upon UV-B radiation^10^. The synthesized 25(OH)D_3_ is hydroxylated by 1α-hydroxylase encoded by the *CYP27B1* gene, and the most active metabolite of vitamin D, 1α,25-dihydroxyvitamin D_3_ [1α,25(OH)_2_D_3_], is yielded in this process^11, 12^. *CYP24A1*, which encodes the vitamin D-deactivating enzyme 24-α hydroxylase, is responsible for inactivating vitamin D metabolites^13^. *CYP24A1* converts 1α,25(OH)_2_D_3_ to 1,24,25(OH)_3_D_3_, which is a less active intermediate^14^. Thus, both *CYP24A1* and *CYP27B1* are members of the cytochrome P450 superfamily, which can regulate 1α,25(OH)_2_D_3_ metabolism by repressing *CYP24A1* and inducing *CYP27B1* through negative feedback loops^15^. Aside from the classic regulatory effects on calcium and phosphate metabolism, 1α,25(OH)_2_D_3_ can also inhibit the proliferation of tumour cells^16^. Several *in vitro* studies have indicated that 1α,25(OH)_2_D_3_ would be able to reduce epithelial cell proliferation, promote differentiation in colon cells, and induce apoptosis in colorectal tumour cell lines^17–19^.

Multiple lines of evidence suggest that genetic factors such as single-nucleotide polymorphisms (SNPs) modify gene expression and consequently influence cancer risk and prognosis. MicroRNAs (miRNAs) are endogenous noncoding RNAs of ~22 nucleotides (nt) in length, which regulate genes by pairing to the 3′-untranslated regions (UTRs) of messenger RNAs (mRNAs) of target genes and specifying mRNA cleavage or repression of protein synthesis^20^. Some 3′-UTR polymorphisms that may be in the vicinity of a miRNA binding site have been reported to interfere with miRNA function and lead to differential gene expression. The SNPs located within miRNA binding sites could thus influence cancer risk and overall survival^21–24^. To date, several studies have focused on the association of polymorphisms in *CYP24A1* and *CYP27B1* with cancer risk^25–27^. However, very little research has addressed the role of polymorphisms in miRNA binding sites. Besides, as is well known that successfully identifying the interactions between genes and dietary factors is important to explore the aetiology of cancer. A more detailed understanding of gene–environment (diet) interaction may thus also generate the information required to develop strategies for diet modification to reduce the incidence of CRC in individuals with specific genetic variants of *CYP24A1* and *CYP27B1*.

Against this background, we carried out this study to investigate whether polymorphisms in the target sites of miRNA in *CYP24A1* and common variation in *CYP27B1* are associated with the risk and prognosis of CRC. We also assessed such effects in terms of their combination and interaction with dietary factors regarding the contribution to the risk of CRC.

## Results

### Characteristics of study subjects

The demographic characteristics of all subjects in this study are summarized in Table 1. The ages (Mean ± SD) of cases and controls were 60.31 ± 11.30 and 57.14 ± 11.21, respectively (*P* < 0.001). Compared with controls, cases presented a lower body mass index (23.26 ± 3.37, *P* < 0.001). Consequently, age and BMI were adjusted in the following multivariate analyses. No significant differences were observed between cases and controls for sex (*P* = 0.685), occupation (*P* = 0.267) and family history of cancer (*P* = 0.102).

**Table 1:** Demographic characteristics of study subjects.

**Variable** ^**a**^	**Case No. (%)**	**Control No. (%)**	***P-*** **value**
	**528**	**605**	
**Age (years)**			**<0.001**
<50	88 (16.67)	152 (25.12)	
≥50 and <60	161 (30.49)	210 (34.71)	
≥60 and <70	157 (29.74)	152 (25.12)	
≥70	122 (23.11)	91 (15.04)	
Mean ± SD	60.31 ± 11.30	57.14 ± 11.21	
**Sex**			0.685
Male	310 (58.71)	348 (57.52)	
Female	218 (41.29)	257 (42.48)	
**BMI** ^b^			**<0.001**
≤18.5	40 (7.59)	35 (5.86)	
>18.5 and ≤23	208 (39.47)	189 (31.66)	
>23 and ≤30	266 (50.47)	330 (55.28)	
>30	13 (2.47)	43 (7.20)	
Mean ± SD	23.26 ± 3.37	24.30 ± 4.21	
**Occupation** ^c^			0.267
Mental worker	134 (25.97)	175 (29.31)	
Physical worker	97(18.80)	121 (20.27)	
Mixed	285 (55.23)	301 (50.42)	
**Family history of cancer**			0.102
No	418 (80.85)	509 (84.55)	
Yes	99 (19.15)	93 (15.45)	

^a^Missing data: occupation, 4 cases; BMI, 1 case, 8 controls; family history of cancer, 11 cases, 3 controls.

^b^BMI, body mass index (weight/height^2^).

^c^Occupation: mental worker (white-collar worker) is the person who perform professional, managerial, or administrative work, such as civil servants, administrative management, scientific research, or education. Physical worker is the person whose job requires manual labor, such as farmer in our questionnaire.

### Polymorphisms of *CYP24A1* and *CYP27B1* and CRC risk

The genotype distributions of all of the four polymorphisms in controls were in accordance with Hardy–Weinberg equilibrium (*P* > 0.05). For rs10877012, GG genotype carriers showed a lower risk of CRC than those with the TT genotype (OR_adjusted_ (OR_adj_) = 0.57, 95% Confidence Interval (CI) = 0.38–0.84) with *P*-values of 0.005 and 0.020 before and after Bonferroni correction, respectively. The recessive model and additive model showed significant results with *P*-values of 0.009 and 0.008, respectively; the recessive model (*P* = 0.036) and additive model (*P* = 0.032) remained significant after multiple testing correction. For rs4646536, the CC genotype reduced the risk of CRC compared with the TT genotype (OR_adj_ = 0.59, 95% CI = 0.40–0.88) with *P*-values of 0.010 and 0.040 before and after Bonferroni correction, respectively; all three genetic models showed significant results with *P*-values of 0.040, 0.023, and 0.010, but only the additive model remained significant after multiple testing correction. We did not observe any noteworthy associations between rs4809957 and rs2762934 in *CYP24A1* and CRC risk (Table 2). Based on the Akaike information criterion (AIC) values, the dominant model was chosen for rs4809957 and the recessive model was chosen for the other three SNPs for use in crossover analysis and multivariate regression analysis.

**Table 2:** Associations between the polymorphisms in *CYP24A1*, *CYP27B1* and the risk of colorectal cancer.

**Genotype** ^**b**^	**Case No. (%)**	**Control No. (%)**	**OR**_**adj**_^**a**^ **(95% CI)**	***P-*** **value**	***P*** ^*^ **value**	**AIC**
***CYP24A1*** **A** > **G (rs4809957)**						
GG	206 (39.31)	230 (38.66)	1.00			
AG	260 (49.62)	295 (49.58)	0.98 (0.76–1.26)	0.867	1.000	
AA	58 (11.07)	70 (11.77)	0.96 (0.64–1.44)	0.844	1.000	
Dominant model			0.98 (0.76–1.25)	0.840	1.000	1508.38
Recessive model			0.97 (0.67–1.42)	0.884	1.000	1508.40
Additive model			0.98 (0.82–1.18)	0.824	1.000	1508.38
***CYP24A1*** **G** > **A (rs2762934)**						
GG	402 (77.76)	467 (78.49)	1.00			
AG	109 (21.08)	120 (20.17)	1.01 (0.75–1.35)	0.972	1.000	
AA	6 (1.16)	8 (1.35)	0.92 (0.31–2.72)	0.885	1.000	
Dominant model			1.00 (0.75–1.34)	0.998	1.000	1499.95
Recessive model			0.92 (0.31–2.71)	0.883	1.000	1499.92
Additive model			0.97 (0.76–1.30)	0.972	1.000	1499.94
***CYP27B1*** **G** > **T (rs10877012)**						
TT	232 (47.44)	230 (42.13)	1.00			
GT	206 (42.13)	233 (42.67)	0.86 (0.66–1.13)	0.282	1.000	
GG	51 (10.43)	83 (15.20)	0.57 (0.38–0.84)	**0.005**	**0.020**	
Dominant model			0.78 (0.61–1.00)	0.053	0.212	1394.31
Recessive model			0.61 (0.42–0.88)	**0.009**	**0.036**	1391.16
Additive model			0.78 (0.65–0.94)	**0.008**	**0.032**	1390.30
***CYP27B1*** **C** > **T (rs4646536)**						
TT	233 (48.04)	229 (42.57)	1.00			
CT	200 (41.24)	229 (42.57)	0.84 (0.64–1.09)	0.190	0.760	
CC	52 (10.72)	80 (14.87)	0.59 (0.40–0.88)	**0.010**	**0.040**	
Dominant model			0.77 (0.60–0.99)	**0.040**	0.120	1375.63
Recessive model			0.64 (0.44–0.94)	**0.023**	0.092	1374.46
Additive model			0.78 (0.66–0.95)	**0.010**	**0.040**	1373.10

^a^OR: odds ratio; CI: confidence interval. OR_adj_: adjusted by age, and BMI.

^b^missing values: rs4809957, 14; rs2762934, 21; rs10877012, 98; rs4646536, 110.

*P* ^*^: *P* values after Bonferroni correction.

### Subgroup analyses

Table 3 displays the results of subgroup analyses for the associations between rs10877012 and rs4646536 polymorphisms in *CYP27B1* and CRC risk. Compared with the wild-type genotype (TT), homozygote variant genotypes (GG of rs10877012; CC of rs4646536) reduced the risk of CRC significantly in the elderly (>60 years) (OR_adj_ = 0.39, 95% CI = 0.21–0.71; OR_adjusted_ = 0.42, 95% CI = 0.23–0.78, respectively), and in females (OR_adj_ = 0.43, 95% CI = 0.23–0.79; OR_adj_ = 0.44, 95% CI = 0.23–0.84, respectively). After adjustment for multiple comparisons, all of these results remained significant. Additionally, no significant result was found in the subgroup analysis by occupation. Only GG of rs10877012 was observed with significant reduced risk of colon cancer (OR_adj_ = 0.42, 95% CI = 0.23–0.78). However, we did not find any significant associations between *CYP24A1* polymorphisms and CRC risk in the subgroup analyses (data not shown).

**Table 3:** Subgroup analyses for the associations between rs10877012, rs4646536 in *CYP27B1* and the risk of colorectal cancer.

**Subgroups**	**rs10877012**			**rs4646536**		
	**TT**	**GT**	**GG**	**TT**	**CT**	**CC**
**Age (years)**						
≤**60**						
Controls/cases	148/119	152/105	45/28	148/119	148/102	44/28
OR_adj_^a^ (95% CI)	1.00	0.87 (0.61–1.24)	0.76 (0.45–1.29)	1.00	0.85 (0.60–1.21)	0.77 (0.45–1.31)
*P* value		0.443	0.312		0.363	0.333
P* value		1.000	1.000		1.000	1.000
**>60**						
Controls/cases	82/112	81/101	38/23	81/114	81/98	36/24
OR_adj_ (95% CI)	1.00	0.85 (0.56–1.29)	0.39 (0.21–0.71)	1.00	0.82 (0.54–1.24)	0.42 (0.23–0.78)
*P* value		0.446	**0.002**		0.344	**0.005**
*P* ^*^ value		1.000	**0.008**		1.000	**0.020**
**Sex**						
**Male**						
Controls/cases	134/130	138/125	43/31	132/131	136/121	43/33
OR_adj_ (95% CI)	1.00	0.96 (0.68–1.36)	0.69 (0.41–1.17)	1.00	0.90 (0.63–1.27)	0.72 (0.42–1.20)
*P* value		0.809	0.172		0.534	0.207
P* value		1.000	0.688		1.000	0.828
**Female**						
Controls/cases	96/101	95/81	40/20	97/102	93/79	37/19
OR_adj_ (95% CI)	1.00	0.71 (0.47–1.09)	0.43 (0.23–0.79)	1.00	0.74 (0.48–1.13)	0.44 (0.23–0.84)
*P* value		0.113	**0.007**		0.161	**0.012**
*P* ^*^ value		0.452	**0.028**		0.644	**0.048**
**Occupation**						
**Mental workers**						
Controls/cases	59/50	68/55	31/21	60/53	65/52	30/20
OR_adj_ (95% CI)	1.00	0.94 (0.56–1.60)	0.70 (0.35–1.39)	1.00	0.85 (0.50–1.44)	0.67 (0.34–1.34)
*P* value		0.830	0.311		0.542	0.254
P* value		1.000	1.000		1.000	1.000
**Physical worker**						
Controls/cases	50/50	44/34	17/9	50/49	42/35	16/9
OR_adj_ (95% CI)	1.00	0.72 (0.39–1.34)	0.38 (0.15–1.00)	1.00	0.82 (0.44–1.52)	0.43 (0.17–1.13)
*P* value		0.304	0.049		0.530	0.087
*P* ^*^ value		1.000	0.196		1.000	0.348
**Mix workers**						
Controls/cases	119/126	116/112	35/20	117/126	117/108	34/22
OR_adj_ (95% CI)	1.00	0.91 (0.63–1.32)	0.51 (0.27–0.94)	1.00	0.83 (0.57–1.21)	0.55 (0.30–1.01)
*P* value		0.611	0.030		0.338	0.054
*P* ^*^ value		1.000	0.120		1.000	0.216
**Site of cancer**						
**Colon**						
Controls/cases	230/84	233/65	83/15	229/84	229/59	80/17
OR_adj_ (95% CI)	1.00	0.72 (0.49–1.06)	0.42 (0.23–0.78)	1.00	0.66 (0.44–0.97)	0.50 (0.28–0.91)
*P* value		0.093	**0.006**		0.036	0.022
*P* ^*^ value		0.372	**0.024**		0.144	0.088
**Rectal**						
Controls/cases	230/135	233/132	83/32	229/136	229/132	80/30
OR_adj_ (95% CI)	1.00	0.95 (0.70–1.29)	0.61 (0.38–0.97)	1.00	0.94 (0.70–1.28)	0.59 (0.37–0.95)
*P* value		0.743	0.037		0.713	0.028
*P* ^*^ value		1.000	0.148		1.000	0.112

Non-mental workers include physical workers and mixed workers.

Note: No significant associations were observed between rs4809957, rs2762934 in *CYP24A1* and CRC risk in subgroup analyses and the results were not listed.

*P* ^*^: *P* values after Bonferroni correction. ^a^OR_adj_: adjusted by age, and BMI.

### Haplotypes of *CYP27B1* and *CYP24A1* and CRC risk

We constructed haplotypes and estimated haplotype frequencies in cases and controls for the four SNPs. The haplotypes with frequencies ≥3% are shown in Supplemental Table 2. Complete linked dimorphism (D′ = 1) was detected for rs4809957 and rs2762934 in *CYP24A1*, while D′ = 0.96 was detected for rs10877012 and rs4646536 in *CYP27B1*. The most common haplotype in *CYP24A1* in cases (64.01%) and controls (63.93%) was G-G. Meanwhile, the most common haplotype in *CYP27B1* in cases (68.16%) and controls (62.18%) was T-C. Compared with those carrying all other types of haplotype, individuals carrying the G-T haplotype showed a decreased CRC risk (OR = 0.80, 95% CI = 0.66–0.97). In contrast, compared with those carrying all other types of haplotype, individuals carrying the T-C haplotype showed an increased CRC risk (OR = 1.25, 95% CI = 1.04–1.51). However, after the Bonferroni correction, no significant association of any haplotype with CRC risk remained.

### Combined and interactive effects of polymorphisms and dietary factors on the risk of CRC

Based on univariate and multivariate analyses of the associations between dietary factors and CRC risk, we found statistically significant results for the consumption of cereals, vegetables, pork, braised fish, soybean, milk, allium vegetables, canned fruit, and overnight meal (Supplemental Table 3). Tables 4 and 5 shows the results of combined and interactive effects of dietary factors and polymorphisms in *CYP24A1* and *CYP27B1*, respectively. For *CYP24A1*, a significant combined effect was only observed for GA + AA genotype carriers in the dominant genetic model combined with the consumption of cereals (≥50 g/week) [OR_genetic&dietary_ (OR_gd_) = 0.41, 95% CI = 0.28–0.60] after Bonferroni correction. No significant interactive effect was observed between dietary factors and polymorphisms in *CYP24A1*.

**Table 4:** Combined and interactive effects between polymorphisms in *CYP24A1* and dietary factors in CRC.

**Dietary factors**	**rs4809957 (dominant model)**			**Interaction**		**rs2762934 recessive model**			**Interaction**	
	**GG**		**GA** + **AA**	**OR**_**i**_ **(95% CI)**	***P***	**GG + GA**		**AA**		
	**OR**_**gd**_ **(95% CI)**					**OR**_**gd**_ **(95% CI)**			**OR**_**i**_ **(95% CI)**	***P***
Cereals (g/week)										
**<**50	1.00	1.01 (0.67–1.53)		0.88 (0.53–1.48)	0.640	1.00	1.17 (0.19–7.23)		0.70 (0.07–7.04)	0.763
≥50	0.45 (0.30–0.68)	**0.41 (0.28**–**0.60)**				0.42 (0.33–0.55)	0.35 (0.08–1.44)			
Overnight meal (times/week)										
>3	1.00	1.05 (0.70–1.56)		1.09 (0.66–1.82)	0.740	1.00	—		—	—
≤3	0.67 (0.45–1.00)	0.65 (0.45–0.93)				0.64 (0.50–0.82)	0.97 (0.31–3.13)			
Allium vegetable (time/week)										
**<**1	1.00	0.95 (0.59–1.52)		1.05(0.60–1.82)	0.876	1.00	0.69 (0.11–4.20)		1.51 (0.16–14.33)	0.721
≥1	0.73(0.47–1.25)	0.72(0.48–1.09)				0.75 (0.57–0.99)	0.78 (0.20–3.01)			
Pork (g/week)										
≥250	1.00	0.97 (0.67–1.42)		1.10 (0.66–1.82)	0.719	1.00	2.28 (0.43–12.12)		8.85 (0.59–132.9)	0.115
**<**250	0.66 (0.44–0.98)	0.58 (0.40–0.84)				0.62 (0.48–0.79)	0.17 (0.02–1.44)			
Milk (time/week)										
≤2	1.00	0.80 (0.58–1.12)		1.68 (0.96–2.93)	0.068	1.00	0.91 (0.26–3.24)		—	—
>2	0.59 (0.38–0.92)	0.80 (0.54–1.17)				0.84 (0.64–1.11)	—			
Vegetable (g/day)										
≤250	1.00	0.92 (0.68–1.24)		1.21 (0.71–2.05)	0.462	1.00	0.75 (0.18–3.21)		1.51 (0.17–13.45)	0.714
>250	1.07(0.72–1.62)	1.20 (0.82–1.74)				1.25 (0.96–1.63)	1.42 (0.28–7.23)			
Canned fruit										
Yes	1.00	0.59 (0.22–1.62)		0.63 (0.22–1.77)	0.378	1.00	—		—	—
No	0.39 (0.16–0.92)	0.37 (0.16–0.87)				0.50 (0.31–0.81)	0.54 (0.16–1.79)			
Braised fish (times/week)										
≥1	1.00	1.12 (0.71–1.77)		1.26 (0.73–2.17)	0.446	1.00	0.49 (0.04–5.53)		0.42 (0.03–6.33)	0.533
**<**1	0.64(0.42–1.00)	0.57 (0.38–0.87)				0.54 (0.41–0.70)	0.63 (0.18–2.12)			
Soybean (times/week)										
>1	1.00	0.97 (0.73–1.29)		1.02 (0.58–1.81)	0.627	1.00	0.74 (0.21–2.69)		0.44 (0.04–4.76)	0.502
≤1	0.81 (0.52–1.26)	0.77 (0.52–1.14)				0.77 (0.58–1.02)	1.29 (0.18–9.36)			

OR_gd_: OR_genetic&dietary_, combined effects of polymorphisms and dietary factors. OR_i_: OR_interaction_, interactive effects of polymorphisms and dietary factors.

Note, we analyzed the combined effects for nine dietary factors and four polymorphisms of CYP24A1 and CYP27B1, the *P* value after Bonferroni correction is 0.05/36 = 0.0014. We analyzed the interactive effects using logistic regression, the significant *P* value of interactive effect is 0.05.

*Bold values indicate significance after Bonferroni correction.

**Table 5:** Combined and interactive effects between polymorphisms in *CYP27B1* and dietary factors in CRC.

**Dietary factors**	**rs10877012 recessive model**		**Interaction**		**rs4646536 (recessive model)**		**Interaction**	
	**TT** + **TG**	**GG**	**OR**_**i**_ **(95% CI)**	***P***	**TT** + **TC**	**CC**		
	**OR**_**gd**_ **(95% CI)**				**OR**_**gd**_ **(95% CI)**		**OR**_**i**_ **(95% CI)**	***P***
Cereals (g/week)								
**<**50	1.00	0.43 (0.23–0.79)	1.76 (0.80–3.84)	0.160	1.00	0.46 (0.25–0.85)	1.77 (0.80–3.90)	0.159
≥50	0.41 (0.319–0.55)	**0.31 (0.19**–**0.52)** ^*^			0.40 (0.30–0.54)	**0.33 (0.20**–**0.54)**		
Overnight meal (times/week)								
>3	1.00	0.44 (0.22–0.85)	0.60 (0.27–1.34)	0.211	1.00	0.49 (0.24–0.97)	0.63 (0.28–1.45)	0.281
≤3	0.62 (0.47–0.81)	**0.45 (0.28**–**0.73)**			0.64 (0.48–0.84)	**0.49 (0.30**–**0.78)**		
Allium vegetable (time/week)								
**<**1	1.00	0.82 (0.42–1.60)	0.62 (0.28–1.41)	0.254	1.00	0.91 (0.45–1.83)	0.60 (0.26–1.39)	0.235
≥1	0.80 (0.59–1.08)	**0.40 (0.24**–**0.68)**			0.78 (0.57–1.06)	**0.43 (0.26**–**0.71)**		
Pork (g/week)								
≥250	1.00	0.68 (0.37–1.24)	1.29 (0.59–2.82)	0.531	1.00	0.63 (0.35–1.15)	1.05 (0.48–2.29)	0.910
**<**250	0.64 (0.49–0.85)	**0.34 (0.20**–**0.57)**			0.64 (0.49–0.85)	**0.39 (0.23**–**0.65)**		
Milk (time/week)								
≤2	1.00	0.57 (0.35–0.93)	1.19 (0.51–2.82)	0.685	1.00	0.58 (0.35–0.95)	1.34 (0.57–3.17)	0.501
>2	0.80 (0.59–1.09)	0.54 (0.27–1.08)			0.78 (0.57–1.06)	0.61 (0.30–1.21)		
Vegetable (g/day)								
≤250	1.00	0.54 (0.34–0.85)	1.51 (0.66–3.50)	0.332	1.00	0.56 (0.36–0.89)	1.58 (0.69–3.65)	0.279
>250	1.15 (0.86–1.54)	0.94 (0.48–1.87)			1.13 (0.84–1.52)	1.01 (0.51–1.98)		
Canned fruit								
Yes	1.00	0.23 (0.04–1.34)	0.33 (0.05–2.07)	0.237	1.00	0.40 (0.07–2.21)	0.57 (0.10–3.29)	0.527
No	0.48 (0.28–0.81)	**0.32 (0.17**–**0.61)**			0.48 (0.28–0.82)	**0.34 (0.18**–**0.64)**		
Braised fish (times/week)								
≥1	1.00	0.43 (0.22–0.86)	0.63 (0.28–1.45)	0.278	1.00	0.40 (0.20–0.80)	0.51 (0.22–1.17)	0.112
**<**1	0.50 (0.37**–**0.68)	**0.34** (**0.21–0.57**)			0.48 (0.36**–**0.65)	**0.37 (0.23–0.62)**		
Soybean (times/week)								
>1	1.00	0.62 (0.41**–**0.94)	1.18 (0.44**–**3.17)	0.740	1.00	0.66 (0.43**–**1.00)	1.17 (0.43**–**3.18)	0.755
≤1	0.81 (0.59**–**1.10)	0.42 (0.18**–**1.01)			0.82 (0.59**–**1.12)	0.46 (0.19**–**1.10)		

OR_gd_: OR_genetic&dietary_, combined effects of polymorphisms and dietary factors. OR_i_: OR_interaction_, interactive effects of polymorphisms and dietary factors.

Note, we analyzed the combined effects for nine dietary factors and four polymorphisms of CYP24A1 and CYP27B1, the *P* value after Bonferroni correction is 0.05/36 = 0.0014. We analyzed the interactive effects using logistic regression, the significant *P* value of interactive effect is 0.05.

^*^Bold values indicate significance after Bonferroni correction.

For rs10877012, significant combined effects were observed for GG genotype carriers in the recessive genetic model combined with the intake of cereals (≥50 g/week, OR_gd_ = 0.31, 95% CI = 0.19–0.52, *P* = 0.000004), overnight meal (≤3 times/week, OR_gd_ = 0.45, 95% CI = 0.28–0.73, *P* = 0.000642), allium vegetables (≥1 times/week, OR_gd_ = 0.40, 95% CI = 0.24–0.68, *P* = 0.000596), pork (<250 g/week, OR_gd_ = 0.34, 95% CI = 0.20–0.57, *P* = 0.00065), canned fruit (No, OR_gd_ = 0.32, 95% CI = 0.17–0.61, *P* = 0.000474), and braised fish (<1 time/week, OR_gd_ = 0.34, 95% CI = 0.21–0.57, *P* = 0.00062). Similar combined effects were found for rs4646536 in *CYP27B1* and dietary factors (as shown in Table 5). However, no significant interactive effects were observed between dietary factors and polymorphisms in *CYP27B1*.

### Polymorphisms, clinical characteristics, and CRC prognosis

The associations of polymorphisms with clinical characteristics in CRC patients are summarized in Supplementary Tables 4 and 5. Significant correlations of rs2765934 in *CYP24A1* with histological classification (*P* = 0.015) and metastasis (*P* = 0.040) were found, as well as of rs10877012 (*P* = 0.036) and rs4646536 (*P* = 0.020) in *CYP27B1* with metastasis.

**Figure 1:**
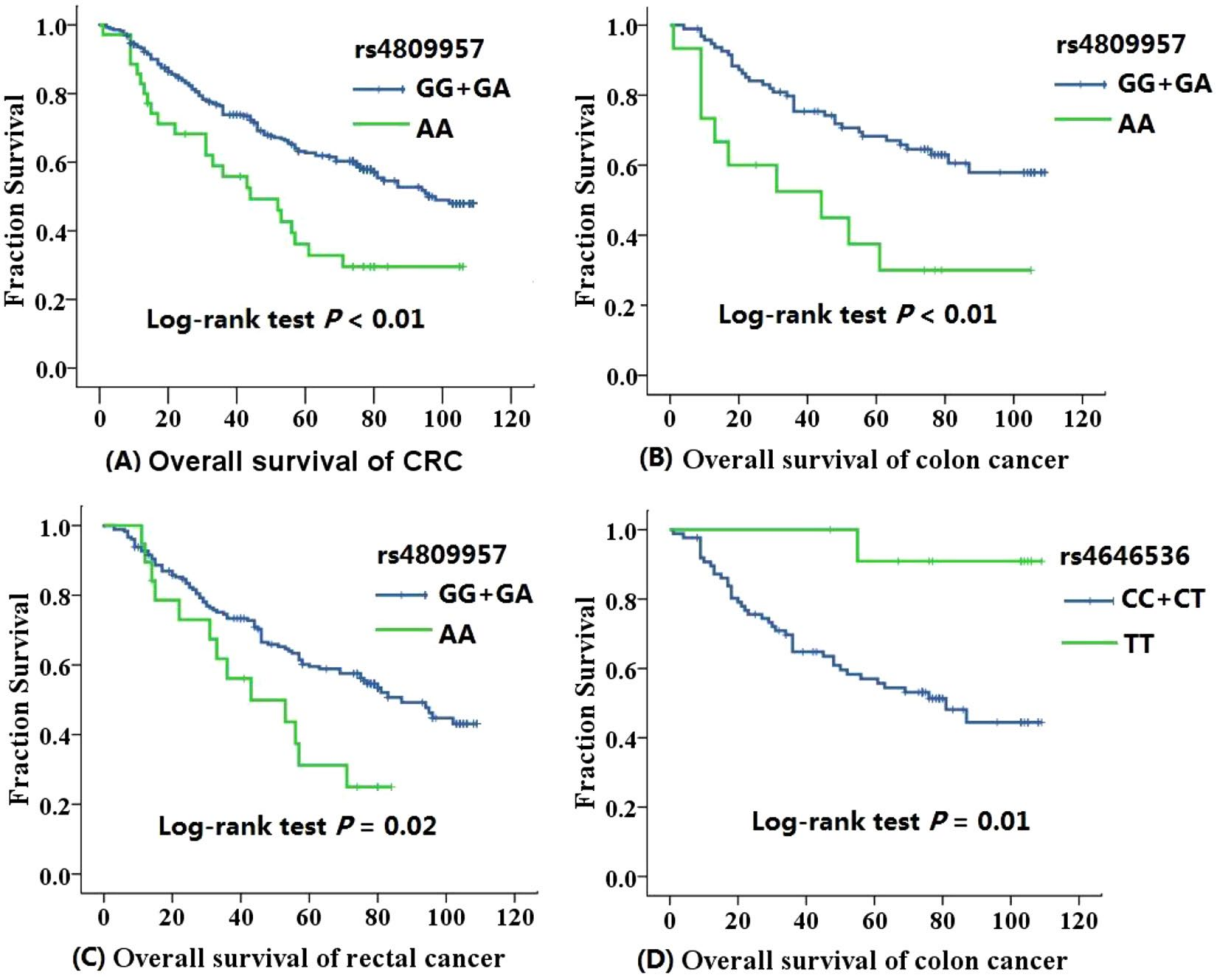
Kaplan-Meier curves of overall survival in CRC (colon and rectal cancer). for *CYP24A1* rs480957 and *CYP27B1* rs4646536 polymorphisms ((**A)**. *CYP24A1* rs480957 in CRC, (**B**) *CYP24A1* rs480957 in colon cancer, (**C**) *CYP24A1* rs480957 in rectal cancer, (**D**) *CYP27B1* rs4646536 in colon cancer).

As shown in Table 6, compared with the GG genotype, AA genotype carriers of rs4809957 polymorphism in *CYP24A1* had worse prognosis (HR = 2.38, 95% CI = 1.30–4.37). Further analysis of the polymorphisms and the prognosis of CRC depending on different sites were conducted. The prognosis of AA (rs4809957) genotype carriers was worse than that of GG + GA carriers in CRC, colon cancer, and rectal cancer (log-rank tests *P* < 0.01, *P* < 0.01, *P* = 0.02, respectively) (Figure 1A–C). For rs4646536, patients who carried the TT genotype had worse prognosis than those with the CC + CT genotypes (log-rank test *P* = 0.01) only in colon cancer (Figure 1D).

**Table 6:** Associations between clinical characteristics, polymorphisms in *CYP24A1*, *CYP27B1* and the prognosis of CRC.

**Prognosis factor**	**Univariate analysis**		**Multivariate analysis**	
	**Hazard ratio (HR)**	***P*** **value**	**Hazard ratio (HR)**	***P*** **value**
	**(95% CI)**		**(95% CI)**	
**Location of primary tumor**		0.404		0.753
Colon	1		1	
Rectum	1.24 (0.87**–**1.77)	0.230	1.00 (0.65**–**1.52)	0.986
Cecum	0.85 (0.30**–**2.37)	0.754	0.67 (0.22**–**2.00)	0.470
**General classification of tumor**		**<0.001**		**0.001**
Protrude type	1		1	
Invasive and	2.02 (1.44**–**2.84)	**<0.001**	1.57 (1.09**–**2.27)	**0.016**
ulcerative type				
Other type	3.66 (1.93**–**6.94)	**<0.001**	3.83 (1.75**–**8.39)	**0.001**
**Histological classification of tumor**		0.782		0.660
Adenocarcinoma	1		1	
Mucinous adenocarcinoma	0.99 (0.53**–**1.84)	0.972	0.81 (0.37**–**1.77)	0.603
Other type	1.34 (0.59**–**3.05)	0.485	1.37 (0.48**–**3.97)	0.559
**Stage of Dukes’**		**<0.001**		**<0.001**
I	1		1	
II	1.71 (0.81**–**3.63)	0.163	1.93 (0.85**–**4.36)	0.112
III	4.27 (2.05**–**8.89)	**<0.001**	8.87 (1.95**–**40.49)	**0.005**
IV	16.47 (7.05**–**38.48)	**<0.001**	40.407 (7.80**–**209.37)	**<0.001**
**Degree of differentiation**		**0.015**		0.691
Low	1		1	
Medium	0.51 (0.34**–**0.78)	**0.002**	0.76 (0.48**–**1.22)	0.254
High	0.73 (0.25**–**2.09)	0.555	0.76 (0.16**–**3.62)	0.727
Unknown	0.80 (0.39**–**1.65)	0.543	0.71 (0.26**–**1.95)	0.506
**Metastasis**		**<0.001**		0.438
No	1		1	
Yes	3.09 (2.20**–**4.33)		0.59 (0.16**–**2.23)	
**Chemotherapy treatent**		0.896		0.206
No	1		1	
Yes	1.02 (0.73**–**1.44)		0.78 (0.54**–**1.14)	
**Anastomat on surgery**		**0.023**		**0.018**
Yes	1		1	
No	1.46 (1.00**–**2.14)	**0.049**	1.80 (1.13**–**2.88)	**0.014**
Undetermined	1.93 (1.10**–**3.39)	**0.023**	1.90 (1.02**–**3.54)	**0.043**
**rs4809957**		**0.008**		**0.017**
GG	1		1	
AG	0.99 (0.69**–**1.42)	0.962	1.20 (0.79**–**1.83)	0.399
AA	2.04 (1.25**–**3.34)	**0.005**	2.38 (1.30**–**4.37)	**0.005**
**rs2762934**		0.370		0.945
GG	1		1	
GA	1.20 (0.81**–**1.78)		1.02 (0.63**–**1.65)	
AA	—		—	—
**rs10877012**		0.888		0.296
TT	1		1	
GT	0.99 (0.69**–**1.41)	0.940	0.58 (0.17**–**2.04)	0.398
GG	0.87 (0.50**–**1.52)	0.628	2.93 (0.41**–**21.05)	0.285
**rs4646536**		0.795		0.226
CC	1		1	
CT	1.05 (0.73**–**1.49)	0.807	1.54 (0.45**–**5.29)	0.497
TT	0.85 (0.48**–**1.51)	0.585	0.24 (0.03**–**1.83)	0.168

Hazard ratio (HR), adjusted by age and BMI.

## Discussion

There is growing evidence that vitamin D reduces the incidence of CRC. At the molecular level, vitamin D suppresses CRC development and growth by affecting cell proliferation, differentiation, apoptosis, and angiogenesis^28^. Polymorphisms located within miRNA binding sites and other gene regions have been reported to play an important role in gene regulation^29^. To date, no study has been conducted on the association between polymorphisms in genes related to vitamin D metabolism (*CYP24A1*, *CYP27B1*) and the risk of CRC in the Chinese population. In our study, it is notable that the GG genotype of the rs10877012 polymorphism decreased CRC risk by about 44%, compared with the TT genotype. Our data also indicated that, compared with the TT genotype, the CC genotype of the rs4646536 polymorphism decreased this risk by about 43%.

Vitamin D insufficiency is common in the elderly. The reduced capacity of the skin to manufacture cholecalciferol^30, 31^ has been evidenced in relation to increased CRC risk in this age group^7, 9^. Based on the subgroup results in our study, compared with the TT genotype, the GG genotype of rs10877012 showed a significant protective role only in elderly participants (>60 years), but not in younger ones (≤60). This difference emphasized the significance of genetic variation on the risk of CRC in the elderly. It indicated that, among the elderly population, individuals carrying the GG genotype exhibit lower activity of *CYP24A1* and a higher level of 1α,25(OH)_2_D_3_, associated with a reduced CRC risk compared with that of individuals with the TT genotype. However, this genetic effect did not significantly modify the CRC risk in younger adults (≤60) because of sufficient cholecalciferol production. Similarly, oestrogen can promote the formation of activated vitamin D by stimulating the secretion of parathyroid hormone^32^. In the total of 433 females in this study, 362 were postmenopausal or on the brink of menopause (aged more than 50); thus, their oestrogen levels had declined significantly. The protective effects of variant genotypes were also highlighted with a decreased risk of CRC only in the female subgroup. Therefore, considering the genetic effects, it should be recommended that the elderly (>60) and postmenopausal women with the TT genotype of rs10877012 and rs4646536 should have more vitamin D supplementation than individuals with GG (rs10877012) and CC (rs4646536) genotypes to obtain the same level of susceptibility to CRC. In addition, solar UV-B radiation is responsible for converting the precursor of vitamin D_3_ (7-dehydrochlolesterol, provitamin D) in the skin into vitamin D_3_^33^. Most humans obtain their required amount of vitamin D (90%–95%) from exposure to sunlight^34^. Previous epidemiological studies demonstrated that exposure to solar UV-B radiation was associated with a decreased risk of colon cancer^35^. Physical worker is considered to have more chance of exposure to solar UV-B because of lots of outdoor work. However, in this study, we did not observe significant results in subgroup analysis by occupation.

Several early studies revealed that CRC could be prevented by dietary fibre^36, 37^. Dietary fibre is a complex carbohydrate derived from plants that escapes digestion in the small intestine and thus reaches the colon. An analysis of data from Europe, North America, and Australia has shown that fibre-rich foods such as cereals are strongly protective against CRC, as are vegetables, while fruit is neutral in this regard^38^. This study produced results consistent with these previous findings, with cereals showing the strongest protective role against CRC. Moreover, significant combined effects were found for cereal intake and SNPs in *CYP24A1* and *CYP27B1*, with ORs ranging from 0.31 to 0.41.

In view of their identified functions associated with adenoma- or carcinoma-related gut microbes, dietary factors have been recognized as major causes of CRC. As a kind of allium vegetables, garlic contains oil-soluble organosulfur compounds such as ajoene, diallyl sulphide, diallyl disulphide, and diallyl trisulphide, whereas onion mainly contains S-propenyl-cysteine sulphoxide, but also other sulphoxides^39^. Researchers have found that diallyl sulphide can penetrate bacterial membranes. Thus, researchers have suggested that the organosulphur compounds in allium vegetables have the potential to be used as antimicrobial agents. In this study, a significantly reduced risk was observed as a combined effect of genetic variants in *CYP27B1* and the consumption of allium vegetables more than once a week. Moreover, bacterial toxins in overnight meal could also cause destruction of the normal gut microbiota and induce chronic gastroenteritis^40^. This is reasonable given the significant protective effects of genetic variants in *CYP27B1* and fewer than three overnight meals per week.

Additionally, a report from Duke Medicine Health News suggested that red meat increases the risk of CRC. It was also reported that diet and lifestyle changes could prevent 64,000 cases of CRC per year in the USA^41^. Another systematic review also indicated that red meat intake is associated with an elevated risk of developing CRC^42^. In this study, compared with the group with the combination of the TT + TG genotype and ≥250 g/week pork intake, the significant combined protective effect of the GG genotype and <250 g/week intake was observed. Especially, this protective effect (OR = 0.34, 95% CI = 0.20–0.57) is much greater than the single main effect of pork (<250 g/week vs. ≥250 g/week, OR = 0.64, 95% CI = 0.49–0.85). It indicated that individuals with the TT + TG genotype should reduce their intake of pork to modify their susceptibility to CRC. Similarly, we found significant combined effects of the GG genotype with no canned fruit intake and with braised fish intake less than once a week. As shown in Table 5, similar combined effects were observed between the two polymorphisms in *CYP27B1* and dietary factors.

The emergence of the field of Molecular pathologic epidemiology (MPE) has emerged as an integrative analysis of exposures, host factors (genetic variants) and dysfunction of cells or organ units^43, 44^. A major value of MPE lies in the provision of a better understanding of heterogeneity in the carcinogenic process and the influences of exogenous and endogenous factors, which should contribute further to personalized prevention and treatment strategies. Based on further validation of the combined effects that we identified in this study, guidelines for dietary intake for individuals with a specific genetic background should be addressed.

There is a paucity of information on the associations of clinical characteristics and 25-OHD level or polymorphisms in genes related to vitamin D metabolism. Four published studies on patients with CRC reported better overall survival among those with higher 25(OH)D_3_ levels than those with lower levels^45–48^. miRNAs are involved in post-transcriptional regulation and can regulate the expression of genes by targeting messenger RNA (mRNA) to degrade or suppress the translation of mRNA^49, 50^. *CYP24A1* catalyses an irreversible and rate-limiting step in the degradation of 1α,25(OH)_2_D_3_^27^. To our knowledge, this is the first study to reveal a significant effect of rs4809957 polymorphism, which is in a miRNA binding site of the *CYP24A1* gene, on the prognosis of CRC patients.

Our study is associated with several potential limitations. First, recall bias may be inevitable when collecting information on dietary factors, although we did our best to minimize it. Second, the frequencies of dietary factors in our questionnaire limited our ability to quantify food intake or calculate the precise intake of vitamins and nutrients. Third, the relatively small size of this study probably reduced the statistical power in the subgroup. Additionally, Bonferroni correction is considered to be a conservative procedure to counteract the problem of multiple comparisons; some ‘real’ effects may have gone undetected in our analysis.

## Conclusions

In conclusion, our study suggested that two polymorphisms in the *CYP27B1* gene are associated with the risk of CRC, particular in the subgroups of the elderly, women, and non-mental workers. Although the interactions had no significance, we observed combined effects of the polymorphisms in *CYP27B1* with dietary factors regarding CRC risk. Moreover, the rs4809957 polymorphism in the *CYP24A1* gene may be an independent predictor of survival in CRC among the population in northeast China. Further epidemiological studies with a large scale and more polymorphisms of genes related to vitamin D metabolism are needed to confirm these findings.

## Methods

### Study subjects

We carried out this study after obtaining written informed consent from the subjects and approval from the Human Research and Ethics Committee of Harbin Medical University. All experiments including all relevant details were performed in accordance with relevant guidelines and regulations.

A case–control study was designed to assess the roles of genetic polymorphisms in *CYP24A1* and *CYP27B1* as well as dietary factors in the risk of CRC. The subjects in this study consisted of 528 patients with primary sporadic colorectal cancer and 605 cancer-free controls (520 hospital-based and 85 community-based). Cases were recruited from the Second and Third Affiliated Hospitals of Harbin Medical University before surgery. Patients were excluded if they suffered from the following diseases: neuroendocrine carcinoma, malignant melanoma, non-Hodgkin’s lymphoma, gastrointestinal stromal tumours, Lynch syndrome, or familial adenomatous polyposis. Controls were recruited from the Second Affiliated Hospital of Harbin Medical University and Hong Qi community of Harbin. Controls with any kinds of cancer or gastrointestinal diseases were excluded. All subjects were enrolled from June 2004 to January 2008. Approximately 5 ml of peripheral venous blood was obtained from all cases either before surgery for the patients or at enrolment for the controls.

A cohort study was also proposed to explore the potential factors associated with the prognosis of CRC patients. For this purpose, among the 528 CRC patients, 317 were followed up from November 2004 to March 2015 via telephone interviews.

### Data collection

All subjects were interviewed face-to-face by trained interviewers using the same questionnaire comprising questions on both demographic characteristics and dietary factors. Demographic data included age, sex, height, weight, nationality, education, marital status, occupation, and family history of cancer. Detailed dietary status in the last year before the diagnosis of disease included the consumption of cereals, allium vegetables, pork, milk, fruit, braised fish, soybean, canned fruit, and overnight meals.

All of the CRC patients were followed up from November 2004 to March 2015 by a telephone call once a year. Clinical information (tumour size and location, general classification, histological and pathological types, Duke stage, degree of differentiation, lymph node metastasis, chemotherapy) was collected from the medical records. Overall survival time was calculated from the date at which patients were diagnosed to the date of their death from any cause; patients who were lost to follow-up, suffered from recurrence, or were still alive at the end of the follow-up were measured as censored data.

### SNP selection and genotyping

We analysed the SNPs in the miRNA binding sites within the 3′-UTR of *CYP24A1* and *CYP27B1* by an extensive search in dbSMR (http://miracle.igib.res.in/polyreg/). Using RNA hybrid (http://bibiserv.techfak.uni-bielefeld.de/rnahybrid/submission.html), the Gibbs free energy [DG, expressed in kilojoules per mole (kJ/mol)] for both wild-type and variant alleles of each SNP was determined; the difference of DG between the two alleles (wild-type allele DG − variant allele DG) was calculated as DDG. The sum of all |DDG|s for each SNP (|DDG tot|) was calculated for predicting the biological impact of the polymorphisms. Three polymorphisms (rs4809957, rs2762934, rs16999067) in target sites of miRNA in *CYP24A1* were predicted, but one (rs16999067) has no allele frequency in the Chinese population; thus, two polymorphic sites (rs4809957 and rs2762934) were selected in this study. There are no polymorphisms in miRNA target sites in *CYP27B1*, but two common polymorphisms (rs10877012 and rs4646536) with a minor allele frequency of more than 5% in the Chinese population were also selected based on previous *in vitro* studies. Detailed information on these four genetic polymorphisms is provided in Supplemental Table 1.

DNA was extracted from leukocytes using the QIAamp DNA Blood Mini Kit, in accordance with the manufacturer’s protocol (Qiagen, Valencia, CA, USA). DNA samples were genotyped using fluorogenic 5′-nuclease assay (TaqMan SNP Genotyping Assay; Applied Biosystems, Foster City, CA, USA) on a Lightcycler® 480|| (Roche, Applied Biosystems) platform. The assay IDs of the probes were C_3120981_30, C_3120982_30, C_25623453_10, and AHCTA6I. A reaction mix of 25 μl contained 10 ng of DNA, 12.5 μl of Universal PCR Master Mix, and 0.625 μl of probe/primer mix. Polymerase chain reaction (PCR) amplification conditions were as follows: an initial step of 95 °C for 10 min, followed by 40 cycles of 92 °C for 15 s and 60 °C for 1 min. We repeated the genotyping for 10% of samples at random.

### Statistical analysis

Categorical variables were tested by chi-square test and continuous variables were tested by two-sample t-test between cases and controls. The genotype distributions in controls were tested for Hardy–Weinberg equilibrium. The AIC statistic was applied to determine the goodness of model fitting. The model with the lowest AIC was considered as the best model and was analysed in subsequent crossover analysis and interaction analysis. To correct for multiple testing, we used the Bonferroni-corrected *P*-value. Haplotypes were evaluated using SHEsis software. We used D′ to assess the extent of linkage disequilibrium (LD) of SNPs in the genome. Univariate and multivariate logistic regression analyses were used to calculate crude and adjusted ORs and 95% CIs. The combined and interactive effects between genetic polymorphisms and dietary factors were estimated by crossover analysis and multivariate logistic regression^51^.

Kaplan–Meier curve and log-rank test were used to assess the influence of genetic variants on overall survival. HRs and corresponding 95% CIs were computed using univariate and multivariate Cox proportional hazard models. Statistical analyses were carried out using SAS, version 9.2 (SAS Institute, Cary, NC, USA). All reported *P*-values were two-sided, and *P* ≤ 0.05 was considered to represent a significant difference.

## Electronic supplementary material

Supplementary Tables

## References

[1] Potter JD (1995). Risk factors for colon neoplasia–epidemiology and biology. Eur J Cancer.

[2] Jemal A (2011). Global cancer statistics. CA Cancer J Clin.

[3] Siegel R, Desantis C, Jemal A (2014). Colorectal cancer statistics, 2014. CA Cancer J Clin.

[4] Jain M (1980). A case-control study of diet and colo-rectal cancer. Int J Cancer.

[5] Potter JD, McMichael AJ (1986). Diet and cancer of the colon and rectum: a case-control study. J Natl Cancer Inst.

[6] Potter JD (1996). Nutrition and colorectal cancer. Cancer Causes Control.

[7] McCullough ML (2003). Calcium, vitamin D, dairy products, and risk of colorectal cancer in the Cancer Prevention Study II Nutrition Cohort (United States). Cancer Causes Control.

[8] Garland CF (1989). Serum 25-hydroxyvitamin D and colon cancer: eight-year prospective study. Lancet.

[9] Wu K, Willett WC, Fuchs CS, Colditz GA, Giovannucci EL (2002). Calcium intake and risk of colon cancer in women and men. J Natl Cancer Inst.

[10] Deeb KK, Trump DL, Johnson CS (2007). Vitamin D signalling pathways in cancer: potential for anticancer therapeutics. Nat Rev Cancer.

[11] Evans SR, Schwartz AM, Shchepotin EI, Uskokovic M, Shchepotin IB (1998). Growth inhibitory effects of 1,25-dihydroxyvitamin D3 and its synthetic analogue, 1alpha,25-dihydroxy-16-ene-23yne-26,27-hexafluoro-19-nor-cholecalcifero l (Ro 25-6760), on a human colon cancer xenograft. Clin Cancer Res.

[12] Jacobs ET (2013). CYP24A1 and CYP27B1 polymorphisms modulate vitamin D metabolism in colon cancer cells. Cancer Res.

[13] Hamamoto H (2006). Structure-function analysis of vitamin D 24-hydroxylase (CYP24A1) by site-directed mutagenesis: amino acid residues responsible for species-based difference of CYP24A1 between humans and rats. Mol Pharmacol.

[14] Jones G, Prosser DE, Kaufmann M (2012). 25-Hydroxyvitamin D-24-hydroxylase (CYP24A1): its important role in the degradation of vitamin D. Arch Biochem Biophys.

[15] Ordonez-Moran P (2005). Vitamin D and cancer: an update of *in vitro* and *in vivo* data. Front Biosci.

[16] Haussler MR (1998). The nuclear vitamin D receptor: biological and molecular regulatory properties revealed. J Bone Miner Res.

[17] Ylikomi T (2002). Antiproliferative action of vitamin D. Vitam Horm.

[18] Diaz GD, Paraskeva C, Thomas MG, Binderup L, Hague A (2000). Apoptosis is induced by the active metabolite of vitamin D3 and its analogue EB1089 in colorectal adenoma and carcinoma cells: possible implications for prevention and therapy. Cancer Res.

[19] Tangpricha V (2001). 25-hydroxyvitamin D-1alpha-hydroxylase in normal and malignant colon tissue. Lancet.

[20] Bartel DP (2004). MicroRNAs: genomics, biogenesis, mechanism, and function. Cell.

[21] Langevin SM, Christensen BC (2014). Let-7 microRNA-binding-site polymorphism in the 3′UTR of KRAS and colorectal cancer outcome: a systematic review and meta-analysis. Cancer medicine.

[22] Saridaki Z (2014). A let-7 microRNA-binding site polymorphism in KRAS predicts improved outcome in patients with metastatic colorectal cancer treated with salvage cetuximab/panitumumab monotherapy. Clin Cancer Res.

[23] Yuan Y (2017). A functional variant at the miRNA binding site in E2F1 gene is associated with risk and tumor HPV16 status of oropharynx squamous cell carcinoma. Molecular carcinogenesis.

[24] Lee AR, Park J, Jung KJ, Jee SH, Kim-Yoon S (2016). Genetic variation rs7930 in the miR-4273-5p target site is associated with a risk of colorectal cancer. OncoTargets and therapy.

[25] Vidigal VM (2017). Genetic polymorphisms of vitamin D receptor (VDR), CYP27B1 and CYP24A1 genes and the risk of colorectal cancer. The International journal of biological markers.

[26] Zeljic K (2012). Vitamin D receptor, CYP27B1 and CYP24A1 genes polymorphisms association with oral cancer risk and survival. Journal of oral pathology & medicine: official publication of the International Association of Oral Pathologists and the American Academy of Oral Pathology.

[27] Fuhrman BJ (2013). Sunlight, polymorphisms of vitamin D-related genes and risk of breast cancer. Anticancer Res.

[28] Rheem DS, Baylink DJ, Olafsson S, Jackson CS, Walter MH (2010). Prevention of colorectal cancer with vitamin D. Scandinavian journal of gastroenterology.

[29] Vosa U, Esko T, Kasela S, Annilo T (2015). Altered Gene Expression Associated with microRNA Binding Site Polymorphisms. PloS one.

[30] Matsuoka LY, Ide L, Wortsman J, MacLaughlin JA, Holick MF (1987). Sunscreens suppress cutaneous vitamin D3 synthesis. J Clin Endocrinol Metab.

[31] MacLaughlin J, Holick MF (1985). Aging decreases the capacity of human skin to produce vitamin D3. J Clin Invest.

[32] Chattar-Cora D, Onime GD, Coppa GF, Valentine IS, Rivera L (1998). Anatomic, age, and sex distribution of colorectal cancer in a New York City Hispanic population. J Natl Med Assoc.

[33] Holick MF (2004). Vitamin D: importance in the prevention of cancers, type 1 diabetes, heart disease, and osteoporosis. Am J Clin Nutr.

[34] Holick MF (2004). Sunlight and vitamin D for bone health and prevention of autoimmune diseases, cancers, and cardiovascular disease. Am J Clin Nutr.

[35] Jongbloet PH (2006). Do sunlight and vitamin D reduce the likelihood of colon cancer? Time for a paradigm shift?. International journal of epidemiology.

[36] Park Y (2005). Dietary fiber intake and risk of colorectal cancer: a pooled analysis of prospective cohort studies. Jama.

[37] Uchida K (2010). Dietary fiber, source foods and colorectal cancer risk: the Fukuoka Colorectal Cancer Study. Scandinavian journal of gastroenterology.

[38] Hill MJ (1997). Cereals, cereal fibre and colorectal cancer risk: a review of the epidemiological literature. European journal of cancer prevention: the official journal of the European Cancer Prevention Organisation (ECP).

[39] Block E (1985). The chemistry of garlic and onions. Scientific American.

[40] Candela, M. *et al*. Inflammation and colorectal cancer, when microbiota-host mutualism breaks. *World J Gastroenterol*, doi:10.3748/wjg.v20.i4.908. (2014). 10.3748/wjg.v20.i4.908 PMC3921544 24574765

[41] Colorectal cancer: red meat increases risk, fiber decreases it. Diet and lifestyle changes could prevent 64,000 cases per year in US *DukeMedicine healthnews* **17**, 5–6 (2011). 27024153

[42] Smolinska K, Paluszkiewicz P (2010). Risk of colorectal cancer in relation to frequency and total amount of red meat consumption. Systematic review and meta-analysis. Archives of medical science: AMS.

[43] Ogino S, Chan AT, Fuchs CS, Giovannucci E (2011). Molecular pathological epidemiology of colorectal neoplasia: an emerging transdisciplinary and interdisciplinary field. Gut.

[44] Ogino S, Stampfer M (2010). Lifestyle factors and microsatellite instability in colorectal cancer: the evolving field of molecular pathological epidemiology. J Natl Cancer Inst.

[45] Fedirko V (2012). Prediagnostic 25-hydroxyvitamin D, VDR and CASR polymorphisms, and survival in patients with colorectal cancer in western European ppulations. Cancer epidemiology, biomarkers & prevention: a publication of the American Association for Cancer Research, cosponsored by the American Society of Preventive Oncology.

[46] Mezawa H (2010). Serum vitamin D levels and survival of patients with colorectal cancer: post-hoc analysis of a prospective cohort study. BMC cancer.

[47] Ng K (2008). Circulating 25-hydroxyvitamin d levels and survival in patients with colorectal cancer. J Clin Oncol.

[48] Ng K (2009). Prospective study of predictors of vitamin D status and survival in patients with colorectal cancer. Br J Cancer.

[49] Reddy SD, Gajula RP, Pakala SB, Kumar R (2010). MicroRNAs and cancer therapy: the next wave or here to stay?. Cancer Biol Ther.

[50] Landi D (2008). Polymorphisms within micro-RNA-binding sites and risk of sporadic colorectal cancer. Carcinogenesis.

[51] Wu YZ (2012). Application of Crossover Analysis-logistic Regression in the Assessment of Gene- environmental Interactions for Colorectal Cancer. Asian Pacific journal of cancer prevention: APJCP.

