# Supplementary material for: Dietary factors and polymorphisms in vitamin D metabolism genes: the risk and prognosis of colorectal cancer in northeast China
Source: Sci Rep. 2017 Aug 18;7:8827. doi: 10.1038/s41598-017-09356-1 (PMC5562792; doi:10.1038/s41598-017-09356-1)
Supplement: Supplementary file 1 — Supplementary Tables [file 41598_2017_9356_MOESM1_ESM.doc]

SUPPLEMENTAL INFORMATION TO

**Dietary factors and polymorphisms in vitamin D metabolism genes: the risk and prognosis of** **colorectal cancer in northeast China**

Chen Gong^1^, Zhiping Long^1^, Yanming Yu^1^, Lin Zhu^1^, Jingshen Tian^1^, Shuo Li^1^, Jing Li^1^, Hongyuan Yu^1^, Qiang Chi^3^, Daxun Piao^4^, Fan Wang^1*^, Yashuang Zhao^1*^, Binbin Cui^2*^

**Supplemental Table 1**

**Information of the polymorphic sites detected in this** study

| **Gene/ dbSNP ID** | **MAF** | **Function** | **Predicted miRNA**  **binding** | **|△△G|b** | **|△△Gtot|c** |
| --- | --- | --- | --- | --- | --- |
| *CYP24A1* rs4809957^a^ | 0.377 | mRNA-untranslated | miR-519e | 13.1 | 56.7 |
|  |  |  | miR-589 | 24.3 |  |
|  |  |  | miR-634 | 19.3 |  |
| *CYP24A1* rs2762934^a^ | 0.169 | mRNA- untranslated | miR-519e | 13.1 | 50.7 |
|  |  |  | miR-589 | 18.3 |  |
|  |  |  | miR-634 | 19.3 |  |
| *CYP27B1* rs10877012 | 0.354 | promoter |  |  |  |
| *CYP27B1* rs4646536 | 0.405 | Intron 6 |  |  |  |

^a^Polymorphisms were selected using dbSMR website (<http://miracle.igib.res.in/polyreg/>).

^b^The |△△G| is the absolute value of the difference for the variant △G minus the wild-type △G using RNAhybrid software.

c|△△Gtot| is the total of the absolute values of △△G (|△△G|).

**Supplemental Table 2**

**Association analyses between the haplotypes of *CYP24A1*, *CYP27B1***

**and the risk of** colorectal cancer

| Haplotypes^a^ | Cases  No.(%) | Controls  No.(%) | OR (95% CI) | *P* value | | *P*^*^ value | LD(D’) |
| --- | --- | --- | --- | --- | --- | --- | --- |
| *CYP24A1* (rs4809957, rs2762934) | |  |  |  |  | | 1.00 |
| A-A | 121 (11.77) | 135 (11.54) | 1.02 (0.79-1.33) | 0.866 | 4.330 | |  |
| A-G | 249 (24.22) | 287 (24.53) | 0.98 (0.81-1.20) | 0.867 | 4.335 | |  |
| G-G | 658 (64.01) | 748 (63.93) | 1.00 (0.84-1.20) | 0.971 | 4.855 | |  |
| *CYP27B1* (rs10877012, rs4646536) | |  |  |  |  | | 0.96 |
| G-T | 295 (30.79) | 377 (35.09) | 0.80 (0.66-0.97) | 0.020 | 0.098 | |  |
| T-C | 653 (68.16) | 668 (62.18) | 1.25 (1.04-1.51) | 0.020 | 0.098 | |  |

Note: Halpotype frequency <0.03 in both control and case has been dropped.

*P*^*^: *P* values after Bonferroni correction.

**Supplemental Table 3**

**Univariate and multivariate analyses for the associations between dietary factors and colorectal cancer risk**

| Variables | Cases  No. (%) | Controls  No. (%) | Univariate analysis | |  | Multivariate analysis | |
| --- | --- | --- | --- | --- | --- | --- | --- |
|  |  |  | OR_adj_  (95% CI) | *P* value |  | OR_adj_  (95% CI) | *P* value |
| Cereals(g/week) |  |  |  |  |  |  |  |
| <50 | 254 (48.20) | 180 (30.05) | 1.00 |  |  | 1.00 |  |
| 50-100 | 118 (22.39) | 139 (23.21) | 0.57 (0.42-0.79) | **0.001** |  | 0.60 (0.40-0.91) | **0.016** |
| 100-200 | 70 (13.28) | 104 (17.36) | 0.43 (0.30-0.63) | **<0.001** |  | 0.36 (0.23-0.58) | **<0.001** |
| >200 | 85 (16.13) | 176 (29.38) | 0.29 (0.21-0.40) | **<0.001** |  | 0.28 (0.18-0.43) | **<0.001** |
| Vegetable(g/day) |  |  |  |  |  |  |  |
| <250 | 72 (13.69) | 47 (7.81) | 1.00 |  |  | 1.00 |  |
| 250-500 | 288 (54.75) | 382 (63.46) | 0.54 (0.35-0.81) | **0.003** |  | 0.45 (0.26-0.79) | **0.005** |
| >500 | 166 (31.56) | 173 (28.74) | 0.72 (0.46-1.12) | 0.141 |  | 0.79 (0.43-1.43) | 0.437 |
| Fruit(times/week) |  |  |  |  |  |  |  |
| Rarely | 96 (18.18) | 90 (14.93) | 1.00 |  |  |  |  |
| <2 | 154 (29.17) | 171 (28.36) | 0.88 (0.61-1.27) | 0.481 |  | 0.85 (0.53-1.38) | 0513 |
| 3-6 | 121 (22.92) | 110 (18.24) | 1.08 (0.73-1.60) | 0.711 |  | 1.16 (0.68-1.96) | 0.586 |
| ≥7 | 157 (29.74) | 232 (38.47) | 0.66 (0.46-0.94) | **0.022** |  | 0.84 (0.51-1.37) | 0.474 |
| Pork(g/week) |  |  |  |  |  |  |  |
| Rarely | 80 (15.47) | 96 (16.52) | 1.00 |  |  | 1.00 |  |
| <250 | 182 (35.20) | 252 (43.37) | 0.89 (0.62-1.28) | 0.524 |  | 0.76 (0.48-1.18) | 0.222 |
| 250-1000 | 195 (37.72) | 203 (34.94) | 1.28 (0.89-1.85) | 0.184 |  | 1.15 (0.72-1.81) | 0.561 |
| >1000 | 60 (11.61) | 30 (5.16) | 3.09 (1.79-5.34) | **<0.001** |  | 3.22 (1.58-6.57) | **0.001** |
| Braised fish(times/week) | |  |  |  |  |  |  |
| <1 | 332 (63.60) | 442 (74.79) | 1.00 |  |  | 1.00 |  |
| ≥1 | 190 (36.40) | 149 (25.21) | 1.78 (1.37-2.32) | **<0.001** |  | 2.30 (1.61-3.26) | **<0.001** |
| Soybean(times/week) |  |  |  |  |  |  |  |
| Rarely | 31 (5.91) | 57 (9.42) | 1.00 |  |  | 1.00 |  |
| <1 | 84 (16.00) | 106 (17.52) | 1.47 (0.86-2.51) | 0.160 |  | 1.40 (0.70-2.79) | 0.340 |
| 2-3 | 185 (35.24) | 270 (44.63) | 1.22 (0.75-2.00) | 0.421 |  | 1.18 (0.62-2.23) | 0.613 |
| ≥4 | 225 (42.88) | 172 (28.43) | 2.37 (1.45-3.89) | **0.001** |  | 2.45 (1.28-4.69) | **0.007** |
| Milk(times/week) |  |  |  |  |  |  |  |
| 0 | 291 (55.53) | 230(48.94） | 1.00 |  |  | 1.00 |  |
| 2-6 | 113 (21.57) | 109 (23.19) | 0.76 (0.55-1.05) | 0.091 |  | 0.61 (0.41-0.90) | **0.013** |
| ≥7 | 120 (22.90) | 131 (27.87) | 0.59 (0.42-0.81) | **0.001** |  | 0.67 (0.45-0.99) | 0.045 |
| Allium vegetable(times/week) | |  |  |  |  |  |  |
| <1 | 160 (30.42) | 138 (22.89) | 1.00 |  |  | 1.00 |  |
| 1-3 | 127 (24.14) | 151 (25.04) | 0.78 (0.56-1.09) | 0.138 |  | 0.58 (0.37-0.89) | **0.013** |
| 4-6 | 85 (16.16) | 135 (22.39) | 0.60 (0.42-0.86) | **0.005** |  | 0.45 (0.28-0.71) | **0.001** |
| ≥7 | 154 (29.28) | 179 (29.69) | 0.81(0.59-1.11) | 0.195 |  | 0.59 (0.38-0.91) | **0.018** |
| Canned fruit |  |  |  |  |  |  |  |
| No | 454 (89.90) | 535 (94.52) | 1.00 |  |  | 1.00 |  |
| Yes | 51 (10.10) | 31 (5.48) | 1.91(1.19-3.08) | **0.008** |  | 2.22 (1.14-4.33) | **0.020** |
| Overnight meal(times/week) | |  |  |  |  |  |  |
| 0 | 106 (20.11) | 135 (22.39) | 1.00 |  |  | 1.00 |  |
| 1-3 | 197 (37.38) | 276 (45.77) | 0.90 (0.66-1.24) | 0.533 |  | 0.84 (0.55-1.28) | 0.405 |
| ≥4 | 224 (42.51) | 192 (31.84) | 1.47 (1.06-2.03) | **0.021** |  | 1.54 (1.00-2.38) | 0.052 |

OR_adj_ : adjusted for age, BMI.

**Supplemental Table 4**

**Association of clinical characteristics with *CYP24A1* polymorphisms in colorectal cancer patients**

| **Characteristic** | **Total (%)** | **rs4809957** | | | ***P***  **value** | **rs2765934 ^a^** | | | ***P***  **value** |
| --- | --- | --- | --- | --- | --- | --- | --- | --- | --- |
|  |  | **AA**  **No.(%)** | **AG**  **No.(%)** | **GG**  **No.(%)** |  | **GG**  **No.(%)** | **AG**  **No.(%)** | **AA**  **No.(%)** |  |
| **Location** | |  |  |  | 0.769 |  |  |  | 1.000 |
| Colon | 110 (34.70) | 44 (40.00) | 51 (46.36) | 15 (13.64) |  | 87 (79.82) | 22 (20.18) | 0 (0.00) |  |
| Rectal | 197 (62.15) | 77 (39.09) | 101 (51.27) | 19 (9.65) |  | 155 (79.90) | 39 (20.10) | 0 (0.00) |  |
| Cecum | 10 (3.16) | 3 (30.00) | 6 (60.00) | 1 (10.00) |  | 8 (80.00) | 2 (20.00) | 0 (0.00) |  |
| **General classification** | |  |  |  | 0.138 |  |  |  | 0.794 |
| Protrude type | 193 (60.88) | 74 (38.34) | 103 (53.37) | 16 8.29) |  | 150 (79.37) | 39 (20.64) | 0 (0.00) |  |
| Invasive and ulcerative type | 109 (34.39) | 45 (41.28) | 49 (44.95) | 15 (13.76) |  | 87 (79.82) | 22 (20.18) | 0 (0.00) |  |
| Other type | 15 (4.73) | 5 (33.33) | 6 (40.00) | 4 (26.67) |  | 13 (86.67) | 2 (13.33) | 0 (0.00) |  |
| **Histological classification** | |  |  |  | 0.124 |  |  |  | **0.015^b^** |
| Adenocarcinoma | 282 (88.96) | 116 (41.14) | 138 (48.94) | 28 9.93) |  | 226 (81.30) | 52 (18.71) | 0 (0.00) |  |
| Mucinous adenocarcinoma | 24 (7.57) | 5 (20.83) | 15 (62.50) | 4 (16.67) |  | 19 (79.17) | 5 (20.83) | 0 (0.00) |  |
| Other type | 11 (3.47) | 3 (27.27) | 5 (45.46) | 3 (27.27) |  | 5 (45.46) | 6 (54.55) | 0 (0.00) |  |
| **Stage of Dukes’** |  |  |  |  | 0.727 |  |  |  | 0.097 |
| Ⅰ | 38 (11.99) | 16 (42.11) | 19 (50.00) | 3 7.90) |  | 33 (86.84) | 5 (13.16) | 0 (0.00) |  |
| Ⅱ | 140 (44.16) | 55 (39.29) | 69 (49.29) | 16 (11.43) |  | 112 (82.35) | 24 (17.65) | 0 (0.00) |  |
| Ⅲ | 119 (37.54) | 42 (35.29) | 62 (52.10) | 15 (12.61) |  | 87 (73.11) | 32 (26.89) | 0 (0.00) |  |
| Ⅳ | 20 (6.31) | 11 (55.00) | 8 (40.00) | 1 5.00) |  | 18 (90.00) | 2 (10.00) | 0 (0.00) |  |
| **Degree of differentiation** | |  |  |  | 0.094 |  |  |  | 0.550 |
| Low | 50 (15.77) | 22 (44.00) | 19 (38.00) | 9 (18.00) |  | 39 (78.00) | 11 (22.00) | 0 (0.00) |  |
| Medium | 244 (76.97) | 97 (39.75) | 123 (50.41) | 24 9.84) |  | 192 (80.00) | 48 (20.00) | 0 (0.00) |  |
| High | 7 (2.21) | 3 (42.86) | 3 (42.86) | 1 (14.29) |  | 7 (100.00) | 0 0.00) | 0 (0.00) |  |
| Unknown | 16 (5.05) | 2 (12.50) | 13 (81.25) | 1 (6.25) |  | 12 (75.00) | 4 (25.00) | 0 (0.00) |  |
| **Metastasis** |  |  |  |  | 0.951 |  |  |  | **0.040^b^** |
| Yes | 179 (56.47) | 71 (39.67) | 89 (49.72) | 19 (10.62) |  | 147 (84.00) | 28 (16.00) | 0 (0.00) |  |
| No | 138 (43.53) | 53 (38.41) | 69 (50.00) | 16 (11.59) |  | 103 (74.64) | 35 (25.36) | 0 (0.00) |  |

^a^The genotyping of 4 DNA samples were not successful for rs27629334.

^b^Bold means statistical significance.

**Supplemental Table 5**

**Association of clinical characteristics with *CYP27B1* polymorphisms in colorectal cancer patients**

| **Characteristic** | **Total (%)** | **rs10877012 ^a^** | | | ***P***  **value** | **rs4646536 ^a^** | | | ***P***  **value** |
| --- | --- | --- | --- | --- | --- | --- | --- | --- | --- |
|  |  | **GG**  **No.(%)** | **GT**  **No.(%)** | **TT**  **No.(%)** |  | **CC**  **No.(%)** | **CT**  **No.(%)** | **TT**  **No.(%)** |  |
| **Location** | |  |  |  | 0.800 |  |  |  | 0.706 |
| Colon | 110 (34.70) | 54 (52.43) | 36 (34.95) | 13 (12.62) |  | 54 (54.55) | 33 (33.33) | 12 (12.12) |  |
| Rectal | 197 (62.15) | 90 (48.13) | 76 (40.64) | 21 (11.23) |  | 92 (49.20) | 75 (40.11) | 20 (10.70) |  |
| Cecum | 10 (3.16) | 4 (40.00) | 4 (40.00) | 2 (20.00) |  | 4 (40.00) | 4 (40.00) | 2 (20.00) |  |
| **General classification** | |  |  |  | 0.274 |  |  |  | 0.399 |
| Protrude type | 193 (60.88) | 84 (46.67) | 70 (38.89) | 26 (14.44) |  | 87 (48.88) | 67 (37.64) | 24 (13.48) |  |
| Invasive and ulcerative type | 109( 34.39) | 54 (50.94) | 43 (40.57) | 9 (8.49) |  | 53 (50.96) | 42 (40.39) | 9 (8.65) |  |
| Other type | 15 (4.73) | 10 (71.43) | 3 (21.43) | 1 (7.14) |  | 10 (71.43) | 3 (21.43) | 1 (7.14) |  |
| **Histological classification** | |  |  |  | 0.489 |  |  |  | 0.541 |
| Adenocarcinoma | 282 (88.96) | 134 (50.19) | 100 (37.45) | 33 (12.36) |  | 134 (50.95) | 98 (37.26) | 31 (11.79) |  |
| Mucinous adenocarcinoma | 24 (7.57) | 11 (47.83) | 11 (47.83) | 1 (4.35) |  | 13 (56.52) | 9 (39.13) | 1 (4.35) |  |
| Other type | 11 (3.47) | 3 (30.00) | 5 (50.00) | 2 (20.00) |  | 3 (30.00) | 5 (50.00) | 2 (20.00) |  |
| **Stage of Dukes’** |  |  |  |  | 0.055 |  |  |  | 0.055 |
| Ⅰ | 38 (11.99) | 15 (42.86) | 16 (45.71) | 4 (11.43) |  | 17 (48.57) | 15 (42.86) | 3 (8.57) |  |
| Ⅱ | 140 (44.16) | 75 (56.82) | 46 (34.85) | 11 (8.33) |  | 74 (57.36) | 45 (34.88) | 10 (7.75) |  |
| Ⅲ | 119 (37.54) | 49 (42.98) | 44 (38.60) | 21 (18.42) |  | 49 (43.36) | 43 (38.05) | 21 (18.58) |  |
| Ⅳ | 20 (6.31) | 9 (47.37) | 10 (52.63) | 0 (0.00) |  | 10 (52.63) | 9 (47.37) | 0 (0.00) |  |
| **Degree of differentiation** | |  |  |  | 0.722 |  |  |  | 0.691 |
| Low | 50 (15.77) | 21 (42.86) | 20 (40.82) | 8 (16.33) |  | 22 (44.90) | 20 (40.82) | 7 (14.29) |  |
| Medium | 244 (76.97) | 119 (51.52) | 86 (37.23) | 26 (11.26) |  | 121 (53.07) | 82 (35.97) | 25 (10.97) |  |
| High | 7 (2.21) | 3 (50.00) | 3 (50.00) | 0 (0.00) |  | 2 (40.00) | 3 (60.00) | 0 (0.00) |  |
| Unknown | 16 (5.05) | 5 (35.71) | 7 (50.00) | 2 (14.29) |  | 5 (35.71) | 7 (50.00) | 2 (14.29) |  |
| **Metastasis** |  |  |  |  | **0.036^b^** |  |  |  | **0.020^b^** |
| Yes | 179 (56.47) | 93 (55.36) | 60 (35.71) | 15 (8.93) |  | 94 (56.97) | 58 (35.15) | 13 (7.88) |  |
| No | 138 (43.53) | 55 (41.67) | 56 (42.42) | 21 (15.91) |  | 56 (42.75) | 54 (41.22) | 21 (16.03) |  |

^a^The genotyping of 17 DNA samples were not successful for rs10877012. The genotyping of 21 DNA samples were not successful for rs4646536

^b^Bold means statistical significance.
